# The Effects of Anthrophony on Song Traits in European Robins (*Erithacus rubecula*)

**DOI:** 10.1002/ece3.73018

**Published:** 2026-02-17

**Authors:** Marzia Golini, Matthew Bell

**Affiliations:** ^1^ School of Biological Sciences, Institute of Ecology and Evolution The University of Edinburgh Edinburgh UK

**Keywords:** bioacoustics, minimum frequency, song complexity, urbanisation

## Abstract

The increasing urbanisation has important impacts on natural soundscapes, through habitat loss and noise pollution (anthrophony), affecting acoustic communication in wildlife. Birds living in cities often adjust their songs to avoid masking by low‐frequency traffic noise, but little is still known about how multiple song traits vary during the day. In this study, we investigated how anthrophony affects song traits in European robins ( *Erithacus rubecula* ) over a 10‐h timeframe by comparing urban and rural populations. We recorded 59 robins in the city of Edinburgh, Scotland, and 54 robins in the Midlothian countryside and compared their songs with linear mixed models. Minimum frequency in rural robins decreased over the day, whereas it remained elevated and constant in urban robins. Urban songs were also longer, less complex and had narrower frequency ranges. These patterns suggest plastic regulation of minimum frequency in response to background noise, anthrophony in urban areas and biophony in rural areas, while suggesting potential constraints on other song traits. Although the fitness consequences of reduced complexity and frequency range remain unclear, behavioural plasticity may help preserve signal reliability in urban habitats.

## Graphical abstract

Our study investigated how anthrophony affected birdsong by comparing European robin songs from urban and rural populations in Midlothian, Scotland. The minimum frequency showed a decreasing pattern in rural robins, but remained higher and constant in urban robins, which also had longer, less complex songs with narrower frequency ranges. Overall, the results suggest a plastic regulation of minimum frequency in response to background noise, although fitness consequences remain unclear.

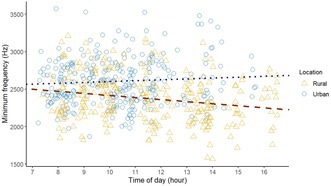

## 1 Introduction

Urbanisation is drastically increasing (Meyer and Turner 1992; Grimm et al. 2008). Between 1950 and 2018 the urban population has risen from 0.75 to 4.22 billion people and is projected to reach 6.68 billion in 2050 (United Nations 2019). Urbanisation is often accompanied by important modifications to the environment, such as deforestation (FAO 2020) and changes in land use (Nuissl and Siedentop 2021) to cropland, pastures or urban settlements (Meyer and Turner 1992). These drastic changes often cause habitat fragmentation (Jaeger 2000), which has severe impacts on the balance and structure of natural ecosystems (Pickett et al. 2001; Marzluff et al. 2012; Li et al. 2022) and disrupts the ecosystem services they provide (Hasan et al. 2020). The main consequence is a rapid decrease in biodiversity (Krauss et al. 2010; Pardini et al. 2017) due to habitat loss (Chase et al. 2020), and the inability of most species to survive in the new hostile environments humans create (Piano et al. 2020). Some species, on the other hand, can either adapt to urban areas by modifying their behaviour (Gil‐Fernandez et al. 2020; Ritzel and Gallo 2020) or cope with human presence (Kittendorf and Dantzer 2021).

Urbanisation can also impact the environment in more indirect ways, via chemical (Saaristo et al. 2018) or acoustic pollution (Slabbekoorn 2019). Anthropogenic noise (anthrophony) comprises sounds made directly or indirectly by humans and is characterised by low frequencies under 2 kHz (Slabbekoorn 2019). In the oceans, noise pollution affects many marine species (Slabbekoorn et al. 2010), such as interfering with whale calls (Foote et al. 2004) or altering behaviour in crabs (Carter 2019). On land there are multiple sources of noise, such as industrial noise (Warrington et al. 2018; Sánchez et al. 2022; Sánchez et al. 2023) or infrastructures (Gil et al. 2015; De Framond and Brumm 2022). Wind turbines also have an impact on songbird behaviour (Zwart et al. 2016; Lehnardt et al. 2024), while in cities, anthrophony interferes with acoustic communication in invertebrates (Morley et al. 2014), amphibians (Sun and Narins 2005; Bee and Swanson 2007) and birds (Halfwerk, Bot, et al. 2011). Overall, the effects of anthrophony can vary greatly based on many different variables, such as exposure length and species (Slabbekoorn 2019).

One of the most common effects of noise pollution is the masking of acoustic signals (Bee and Swanson 2007). Acoustic signals are essential for many species, as they contain information for survival and reproduction (Gerhardt and Bee 2007; Ladich and Winkler 2017; Amorim 2023), and signal masking can thus have significant repercussions on an individual's fitness (Catchpole and Slater 2008; Tennessen et al. 2024; Zhou et al. 2025). Selection has therefore shaped acoustic signals to maximise transmission efficiency within a given environment, as stated by the Acoustic Adaptation Hypothesis (AAH; Morton 1975; Ey and Fischer 2009; Hardt and Benedict 2021). The AAH predicts that signal structure evolves in response to the acoustic properties of the environment. For example, lower frequencies are favoured in closed environments with increased reverberation, such as dense forests (Wiley 1991, 2006), while open spaces, due to the increased attenuation, favour higher frequency signals. In an urban environment characterised by persistent, predominantly low‐frequency anthropogenic noise, the acoustic properties are altered due to the increased masking, which can disrupt acoustic communication (Cho et al. 2025).

Acoustic communication, defined as the transmission of information between individuals through sound signals, is one of the main forms of communication for birds (Catchpole and Slater 2008; Kroodsma and Miller 2020), with each species developing unique songs and calls to allow individuals to communicate with each other (Kumar 2003). Songs are shaped by morphological traits, such as body mass or bill structure and size (Derryberry 2009; Demery et al. 2021), and can vary in frequency and patterns (Catchpole and Slater 2008). Songs play an important role in territory defence (de Kort et al. 2009) and mate selection (Searcy 1992), and for such reasons are often under selection (Mikula et al. 2021). Songs are also shaped by the environment (AAH; Boncoraglio and Saino 2007; Derryberry 2009; Narango and Rodewald 2016).

Although many bird species manage to live and thrive in cities (Bonier et al. 2007; Patankar et al. 2021), acoustic pollution still affects them (Halfwerk, Holleman, et al. 2011) by interfering with the low frequency elements of their songs (Halfwerk, Bot, et al. 2011).

In response to this masking problem, urban birds have modified their song to maximise transmission efficiency under altered acoustic conditions, either by changing their singing time (Fuller et al. 2007; Bermúdez‐Cuamatzin et al. 2020) or by increasing the fundamental frequency of their song (Brumm 2006). The latter has now been observed in many species, including great tits (Slabbekoorn and Peet 2003), song sparrows (Wood and Yezerinac 2006), blackbirds (Nemeth and Brumm 2009) and silvereyes (Potvin et al. 2011). Studies have also shown that there are distinct differences between urban and rural environments also in song length and complexity (Table 1).

**TABLE 1:** Summary table of a selection of studies which focused on analysing differences in song traits between urban and rural environments across different bird species.

Song trait	Urban	Rural	Species
Minimum frequency	High	Low	Blackbird ( *Turdus merula* ; Nemeth and Brumm, 2009); European robin ( *Erithacus rubecula* ; Montague et al., 2013); Great tit ( *Parus major* ; Mockford & Marshall, 2009); Silvereye ( *Zosterops lateralis* ; Potvin et al., 2011)
Maximum frequency	High	High	European robin ( *Erithacus rubecula* ; Montague et al. 2013)
Frequency range	Narrow	Wide	American robin ( *Turdus migratorius* ; Seger‐Fullam et al. 2011); European robin ( *Erithacus rubecula* ; Montague et al. 2013); Northern cardinal ( *Cardinalis cardinalis* ; Seger‐Fullam et al. 2011)
Length	Short	Long	Blackbird ( *Turdus merula* ; Nemeth and Brumm, 2009); European robin ( *Erithacus rubecula* ; Montague et al., 2013); But see: oriental magpie‐robin ( *Copsychus saularis* ; Hill et al., 2018)
Complexity	Simple (less syllables)	Complex (more syllables)	Blackbird ( *Turdus merula* ; Nemeth and Brumm, 2009); European robin ( *Erithacus rubecula* ; Montague et al., 2013); But see: song thrush ( *Turdus philomelos* ; Deoniziak and Osiejuk, 2019)

The European robin ( *Erithacus rubecula* ) is a small insectivorous passerine member of the Muscicapidae family. Females and males are similar, with brownish‐grey plumage and characteristic orange breast. It is a resident in the United Kingdom all year round (P. Lack 2010), singing from dawn until dusk (Da Silva et al. 2014). Outside of the breeding season, both males and females aggressively sing to keep intruders out of their territory (D. Lack 1939; East 1982; Chantrey and Workman 1984; Dudouit et al. 2022). Males are the only ones who sing during the breeding season to attract females (D. Lack 1939), and their territory can extend up to ~1.31 ha (Adriaensen and Dhondt 1990). The territorial song comprises low‐frequency elements below 3.3 kHz (Zwart et al. 2016). By the Acoustic Adaptation Hypothesis, these low‐frequency elements are therefore expected to be particularly vulnerable to masking by urban noise and therefore subject to strong selection or plastic modification, making European robins an ideal species to study the effects of urban noise.

**FIGURE 1:**
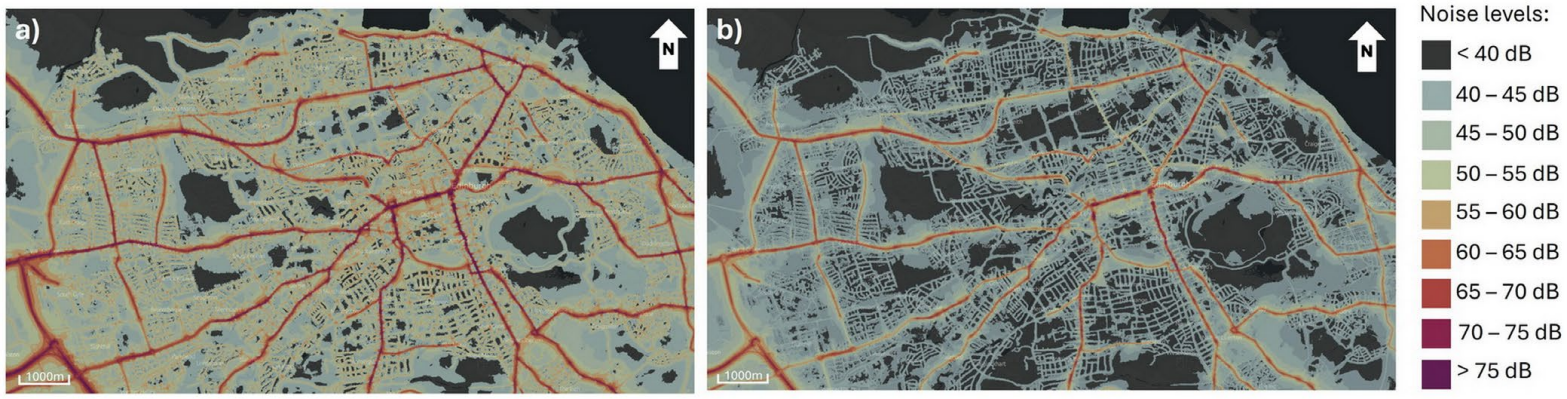
Map of Edinburgh, Scotland, UK, showing road noise (a) during the day and (b) at night. Note the higher level of noise during the day. Maps were taken from https://noise.environment.gov.scot/noise‐map.html and modified in PowerPoint.

In this study, we will look at how anthropogenic noise affects song characteristics in robins living in Edinburgh city and the ones living in the Midlothian countryside across a 10‐h timeframe. The soundscapes of these two areas are very different, affected by structural features and composition of ambient sounds. Edinburgh city, characterised by more open habitats, is dominated by relatively constant daily anthropogenic noise, primarily caused by road traffic and public transport, although noise levels decrease at night (Figure 1). In contrast, outside the city, noise is mainly concentrated near major roads and urban centres. Away from roads, the countryside, with denser, closed‐canopy vegetation, is characterised by a generally quieter soundscape, in which streams, wind and biological sources constitute the main contributors to background noise. Despite not being able to account for structural differences between the two environments, since robins have been shown to respond to experimental noise treatments (Montague et al. 2013), we can expect their song to differ between the two soundscapes, although daily patterns remain understudied. We will be mainly focusing on minimum and maximum frequencies, frequency range, as well as song length and complexity. Based on previous studies (Table 1), we predict that urban robins will exhibit higher minimum frequencies and modified song length and complexity compared to rural individuals.

## 2 Methods

### 2.1 Sample Sites

**FIGURE 2:**
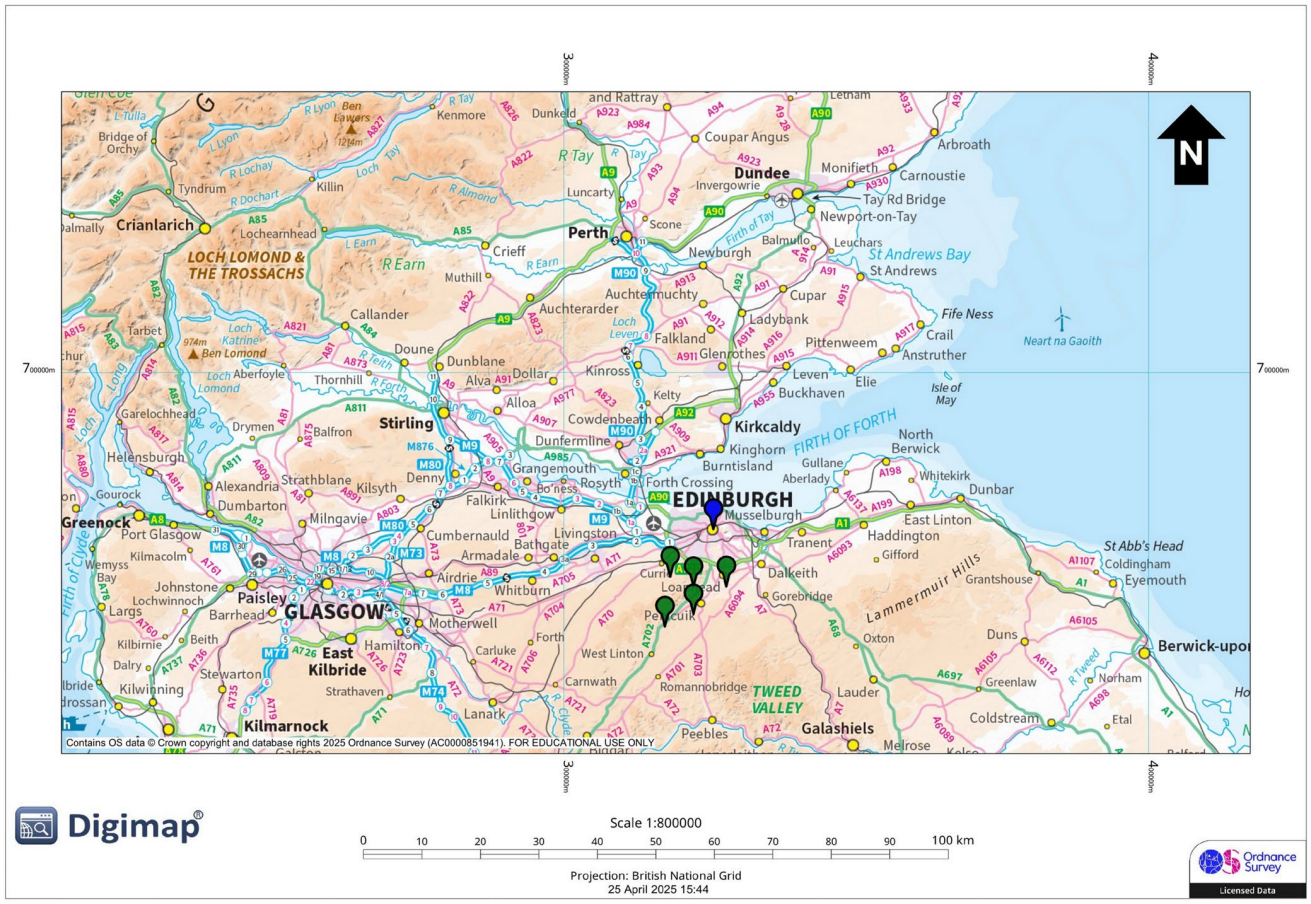
Map of collection sites across Scotland, UK. The blue marker is the urban collection site (Edinburgh city); the green markers are the rural collection sites (Midlothian).

We collected the data between 16 February and 18 March 2025. The sampling was divided into two categories: urban, within the city of Edinburgh, and rural, forest areas within Midlothian (Figure 2). Sites not only differed in anthrophony levels, but also in floral and avian communities (Saha 2024; personal observations).

**FIGURE 3:**
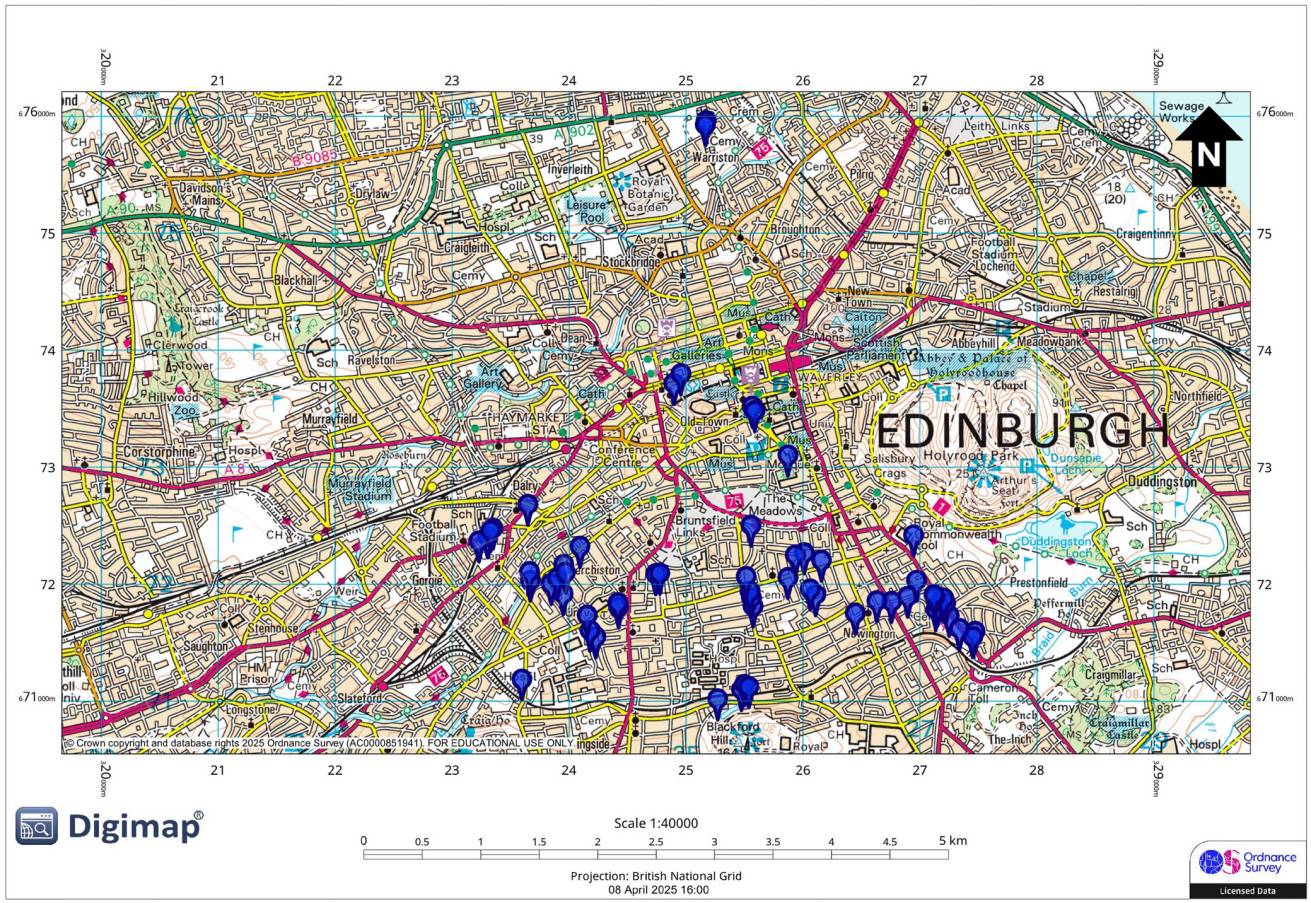
Map of Edinburgh City (Urban environment). Blue markers indicate individual recorded robins.

**FIGURE 4:**
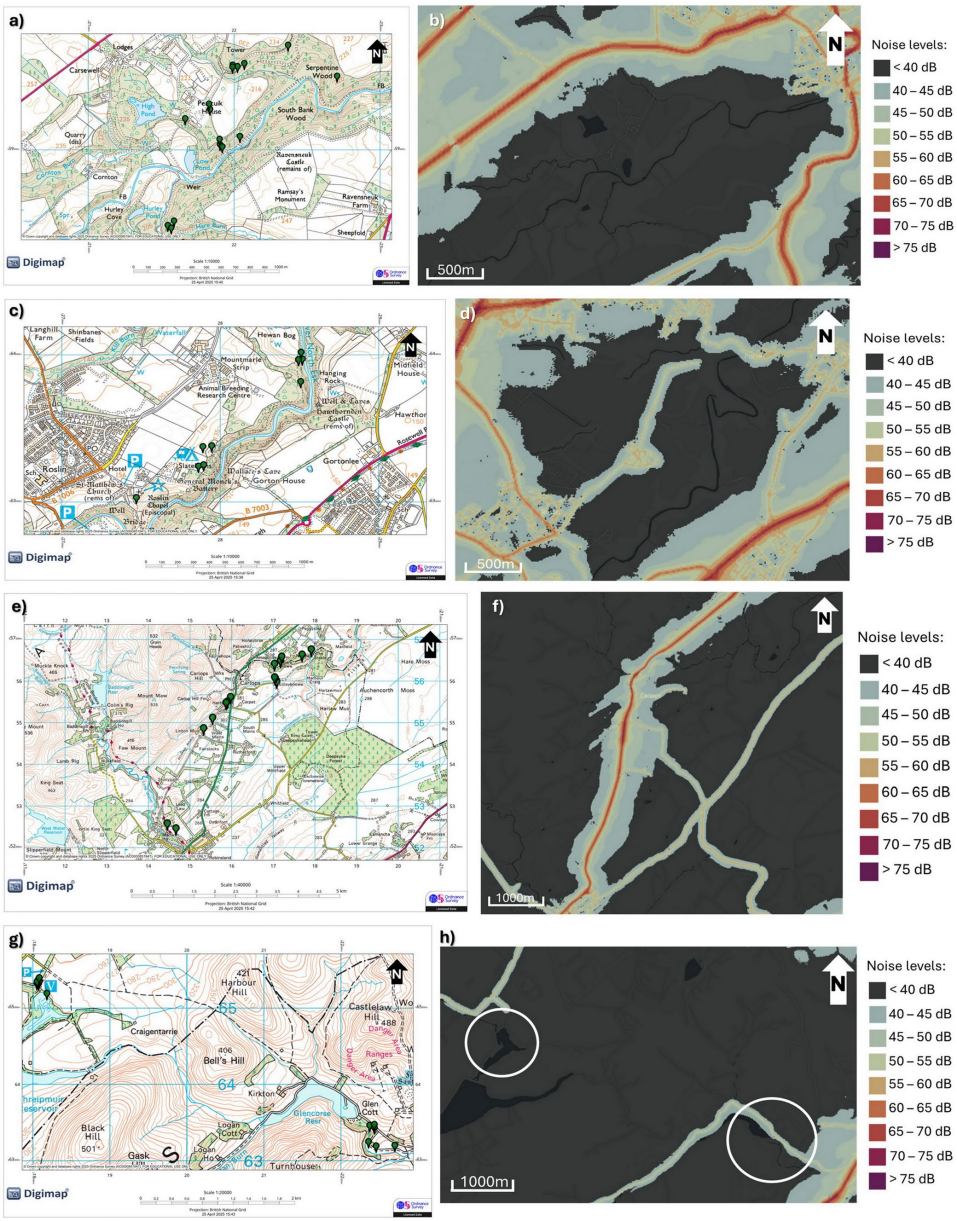
Maps of rural sites in the Midlothian. On the left, collection maps: Green markers indicate individual recorded robins. On the right, maps of general daytime road noise (not at recording times). (a, b) Penicuik Estate. (c, d) Rosling Glen Country Park. (e, f) Newhall Estate. (g) Pentlands Hills Regional Park (bottom right: Glencorse Burn; top left: Harlaw Reservoir). (h) Pentlands Hills Regional Park (white circles around Glencorse Burn and Harlaw Reservoir). Note how each sampling site is quiet. Noise maps were taken from https://noise.environment.gov.scot/noise‐map.html and modified in PowerPoint.

Urban sites were located within Edinburgh city (55.9533° N, 3.1883° W; Figure 3) in parks < 0.5 km^2^ and along streets with continuous anthropogenic noise from road traffic. Rural sites were selected using OS Digimap (https://digimap.edina.ac.uk/) in the Midlothian forest areas with minimal anthropogenic noise, verified using Scotland's Noise maps (< 40 dB background; https://noise.environment.gov.scot/noise‐map.html; Figure 4b,d,f,h). Sites included Penicuik Estate (Figure 4a), Roslin Glen Country Park (Figure 4c), Newhall Estate (Figure 4e), and two woodland sites in Pentland Hills Regional Park (Glencorse Burn and Harlaw Reservoir; Figure 4e).

Anthropogenic noise was not quantified directly at recording sites. Instead, sites were categorised as urban or rural based on habitat structure and on presence or absence of traffic noise. This does not capture fine‐scale variation in anthrophony noise levels within the environments, and any differences in song characteristics should therefore be interpreted as responses to contrasting soundscapes rather than to specific noise intensities.

### 2.2 Sampling Protocol

We sampled rural and urban sites on alternating days to minimise seasonal effects in singing behaviour (Sierro et al. 2017). Sampling was done during daylight hours (0700–1700), as robins sing throughout the day (Da Silva et al. 2014), and with suitable weather conditions (no precipitation, wind ≤ 2 Beaufort; Wood and Yezerinac 2006). Each site was surveyed once along a pre‐defined route to minimise the risk of re‐sampling individuals.

At each site we listened for spontaneously emitted songs, confirming bird identification with the Merlin Bird ID app (Cornell Lab of Ornithology, USA), with additional visual confirmation when possible. We recorded 1 min (Metcalf et al. 2023; Common et al. 2025) of spontaneous song with a Sennheiser ME66 shotgun microphone with a K6 powering module (Sennheiser 2004) connected to a Marantz Professional PMD660 Solid State recorder (D&M Holdings Inc., 2008; sample frequency: 44.1 kHz, resolution: 16 bit, .wav format). The microphone was mounted on a Rycote pistol grip with a fur windshield and directed toward the focal bird. The time and GPS coordinates of each recording were immediately logged using the GaiaGPS app (https://www.gaiagps.com/).

Robins would sometimes sing in proximity to one another on the boundaries of their respective territories, and their songs never overlapped. We used a combination of microphone angling and verbal commentary to identify which songs on the recording belonged to the focal bird and should be analysed.

### 2.3 Acoustic Analysis Protocol

Each recording was renamed with U (urban) or R (rural), followed by the robin's number. All songs were numbered in the order they appeared in the recording.

**FIGURE 5:**
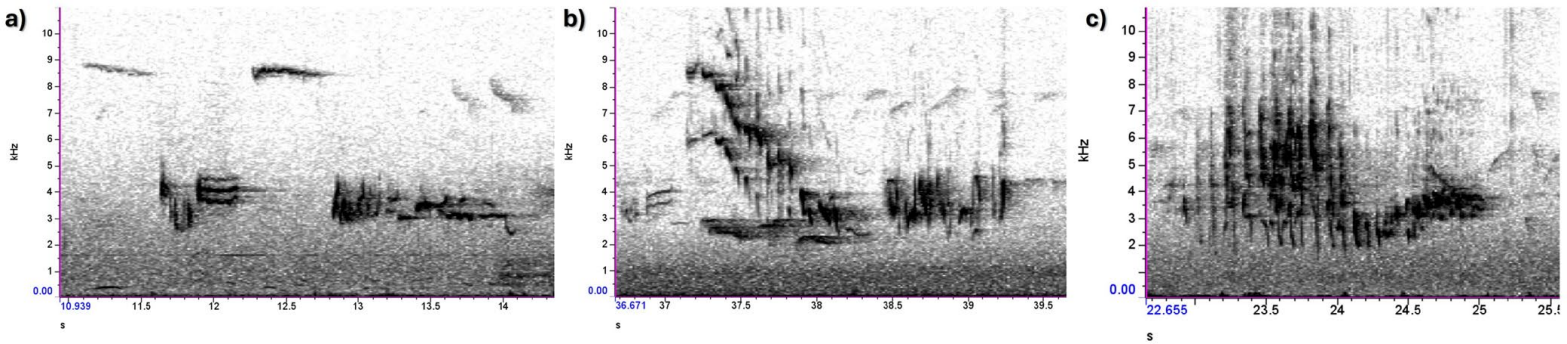
Spectrograms of the three categories of songs in European robins: (a) Up‐down—the number of elements could vary from two to nine (here: R50, song 2—four elements). (b) Slur (here: R1, song 5—slur + down element). (c) Mix (here: R8, song 5). Spectral parameters: Hann window, Brightness = 50, Contrast = 70, Focus = 1300. N.B. Since there is no official robin song classification, we gave a name to these three categories.

After visual and aural examination, we categorised the songs into three types: up‐downs, songs characterised by defined alternating high (up) and low (down) elements (Figure 5a); slurs, songs containing a cascade of slurring notes (often descending), sometimes followed or preceded by up and/or down elements (Figure 5b); and mixes, songs with no clear structure, often sort of a twitter or a warble (Figure 5c).

Up‐down songs were the predominant type (63% of total songs), had clear margins and distinct syllable structure. All robins had at least 2 up‐down songs, while the other two types were not always present. For this reason we only used the up‐down songs for the analysis.

**FIGURE 6:**
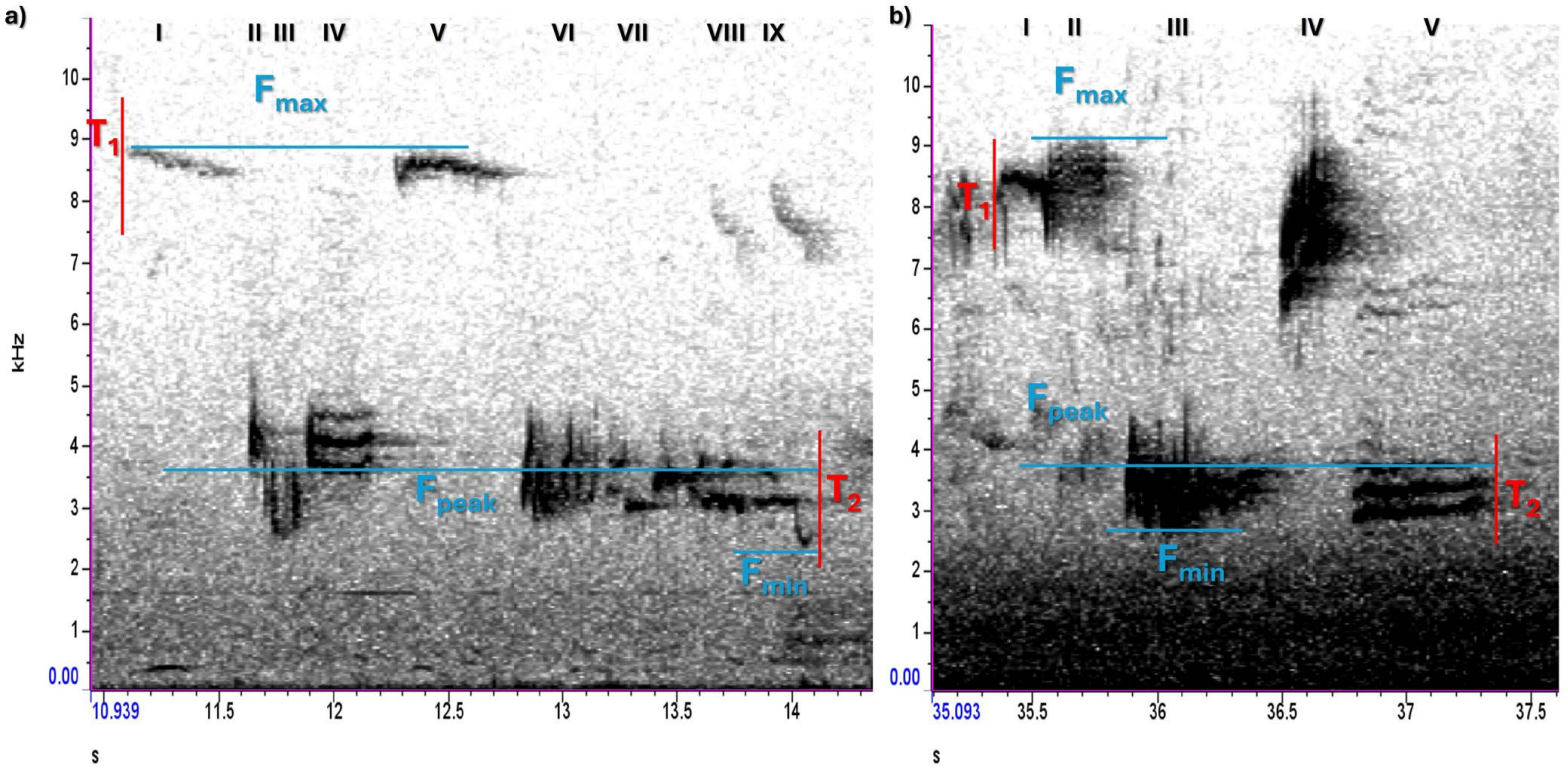
Spectrograms of two up‐down European robin songs in: (a) Rural environment (here: R50, song 2). (b) Urban environment (here: U38, song 6). Song measurements were automatically measured by Raven Pro: Minimum frequency (F_min_), maximum frequency (F_max_), frequency range (F_max_–F_min_) and song length (T_2_–T_1_). Syllables were counted as individual notes through visual and aural analysis. Notice the presence of more background noise in the low frequencies caused by traffic in the urban sample. Spectral parameters: Hann window, Brightness = 50, Contrast = 70, Focus = 1300.

We processed the recordings with the Raven Pro 1.6.5 software (Cornell Lab of Ornithology, USA), using the spectrogram view (Spectral parameters: Hann window, Brightness = 50, Contrast = 70, Focus = 1300) for the analysis. Song length, minimum and maximum frequencies and frequency range were automatically calculated by the software when highlighting the song, and we manually counted the number of syllables via aural and visual analysis (Figure 6). In this analysis, syllables were considered as the number of notes in a song.

### 2.4 Re‐Recording Experiment

To test for background noise dependent measurement error (Zollinger et al. 2012), we conducted a re‐recording experiment on rural recordings (Verzijden et al. 2010). We randomly selected three recordings for each rural location (i.e., a total of 12 recordings), and we created an urban noise track by stitching together song‐free snippets from urban recordings. All recordings were processed in the Audacity v.3.7.7 software and RMS‐normalised to −20 dBFS to control for variation in recording distance and microphone orientation. Each rural audio was then re‐recorded within Audacity with and without the urban noise track (sample frequency: 44.1 kHz, resolution: 16 bit, .wav format). We analysed the new recordings in the Raven Lite 2.0.5 software, with the same spectral parameters used for the previous analysis, measuring the minimum frequencies of all up‐down songs in the recording.

### 2.5 Statistical Analysis

The data were analysed in R v.4.4.1. Each song trait was fit as the response variable in a linear mixed model (LMM).

All models had ‘Location’ as the main predictor variable, while ‘Time’ was included as a fixed effect to account for noise levels throughout the day.

‘Robin ID’ was added as a random effect to account for any individual variation, as all up‐down songs from each robin were included in the analysis.

The interaction between ‘Location’ and ‘Time’ variables was kept only in the minimum frequency model, and it was dropped from all other traits models because it was not significant. For the re‐recording experiment, minimum frequency was analysed with a linear mixed model. ‘Treatment’ was the main predictor variable (no‐noise or with the noise track), and ‘Robin ID’ was included as a random effect.

## 3 Results

We recorded a total of 59 urban and 54 rural robins. All robins contained at least two up‐down songs. Up‐down songs represented more than half of the songs in both urban and rural robins (66% and 60% respectively), for a total of 302 urban songs and 270 rural songs.

### 3.1 Song Traits Between Rural and Urban Populations

**FIGURE 7:**
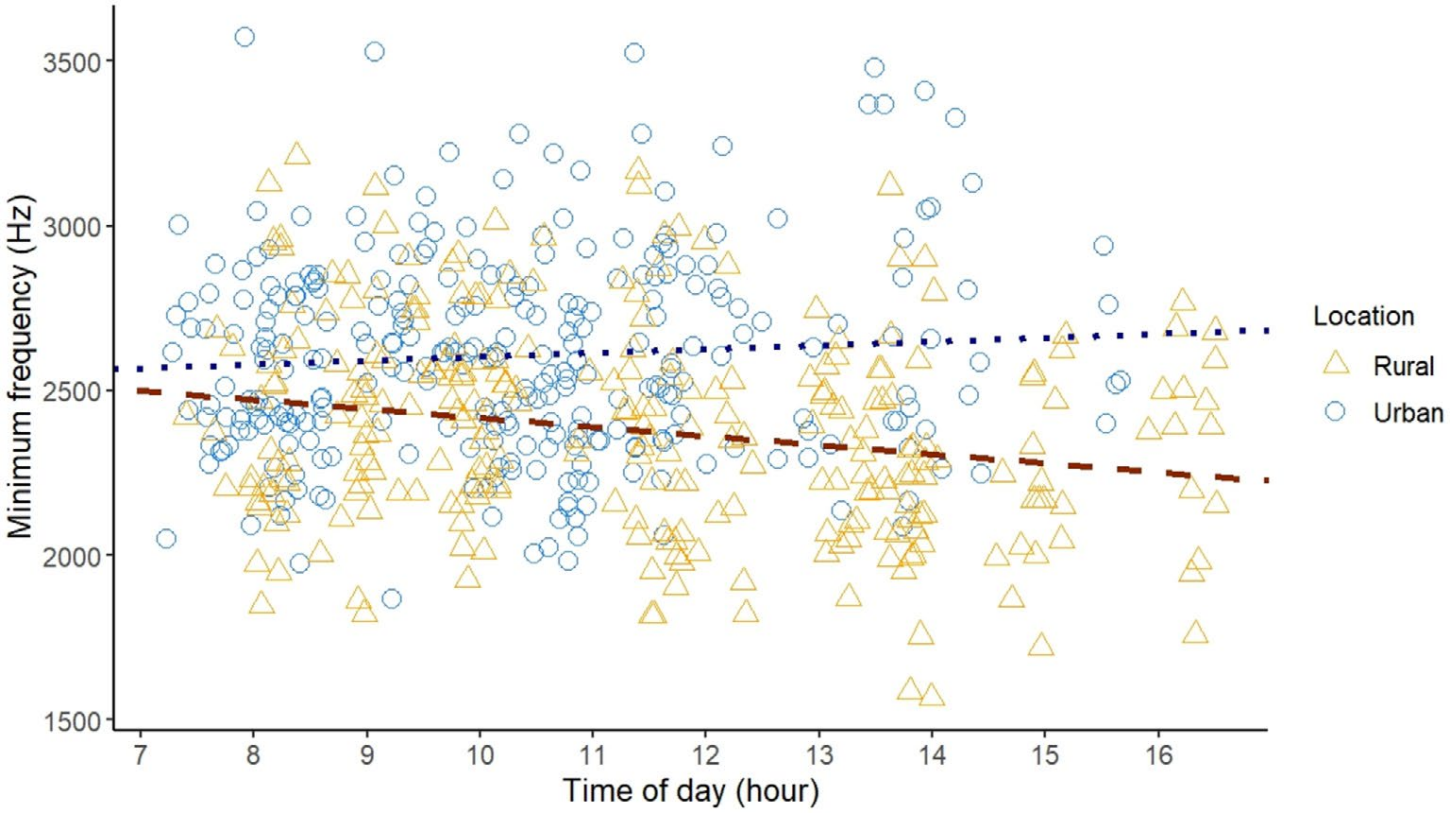
Scatterplot of the minimum frequency of rural (orange triangles) and urban (blue circles) robins against time, with regression lines of the minimum frequency in rural (dashed, dark orange) and urban (dotted, dark blue) environments.

Minimum frequency decreased throughout the day in rural robins (−27.5 Hz per unit time, 95% CI [−47.4, −7.6]), but remained constant in urban robins (+11.6 Hz, 95% CI [−12.4, 35.5]; see Table 2; Figure 7).

**TABLE 2:** Output from a LMM investigating the effects of urbanisation on minimum frequency.

Model terms	Estimate	Std. Error	Df	*t*	*p*
Intercept (Rural)	2691.66	115.48	106.64	23.309	< 2e‐16 ***
Urban	−205.74	170.48	99.74	−1.207	0.2304
Time	−27.51	10.02	105.95	−2.745	0.0071 **
Location:Time	39.09	16.69	100.34	2.491	0.0144 *

Random terms	Variance
Robin ID	13,626
Residuals	84,909

*Note:* Data are from 572 songs recorded from 113 robins. Robin identity was fitted as a random term. Asterisks were reported as shown in the *R* summary view with: *p < 0.05. **p < 0.01. ***p < 0.001.

**FIGURE 8:**
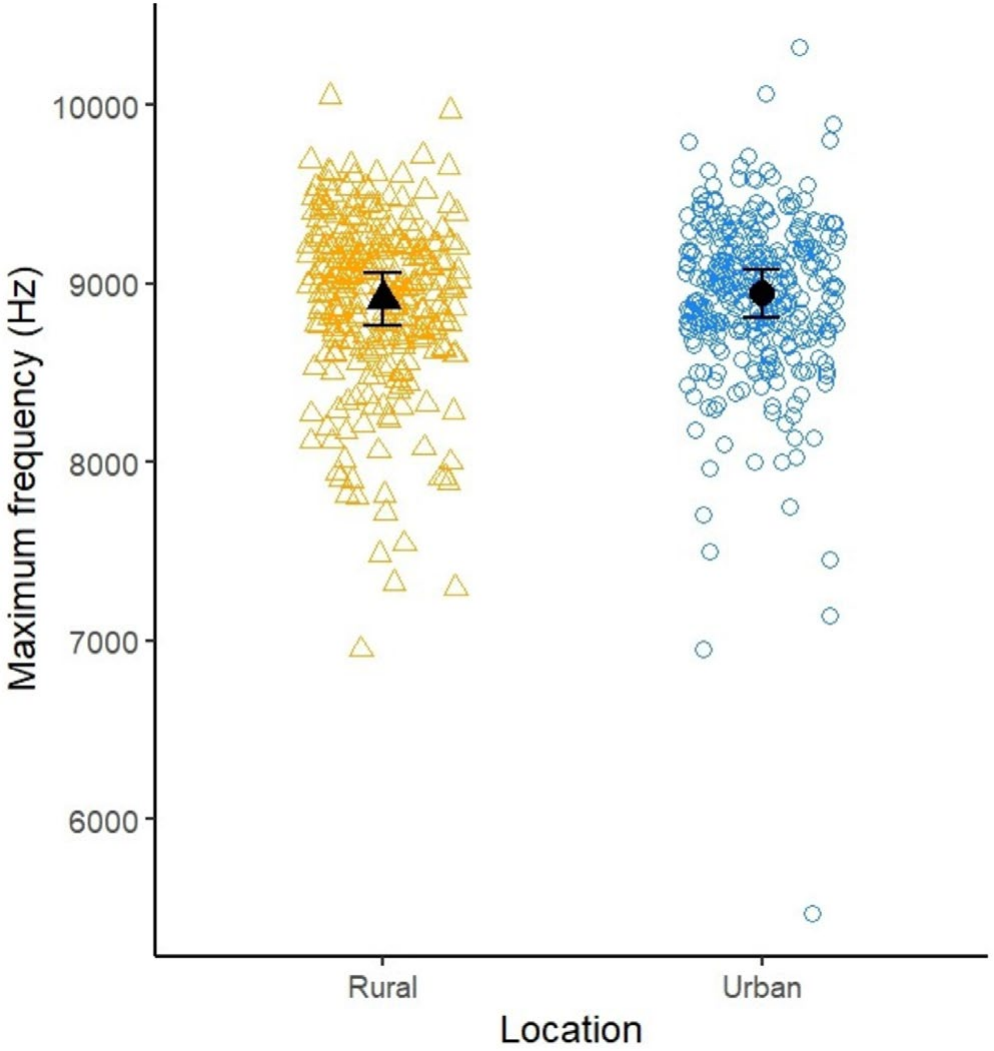
Scatterplot of the maximum frequency of rural (R; orange triangles) and urban (U; blue circles) robins. Open datapoints are raw data, black datapoints are the mean ± s.e. estimated from the model.

There was no difference in the maximum frequency of rural and urban robins (rural—urban: −27.8 Hz, 95% CI [−139, 83.1]; see Table 3; Figure 8).

**TABLE 3:** Output from a LMM investigating the effects of urbanisation on maximum frequency.

Model terms	Estimate	Std. Error	Df	*t*	*p*
Intercept (Rural)	8914.135	146.966	99.583	60.666	< 2e‐16 ***
Urban	27.819	55.947	98.774	0.497	0.620
Time	−2.094	12.564	99.838	−0.167	0.868

Random terms	Variance
Robin ID	44,960
Residuals	181,559

*Note:* Data are from 572 songs recorded from 113 robins. Robin identity was fitted as a random term. Asterisks were reported as shown in the *R* summary view with: ***p < 0.001.

**FIGURE 9:**
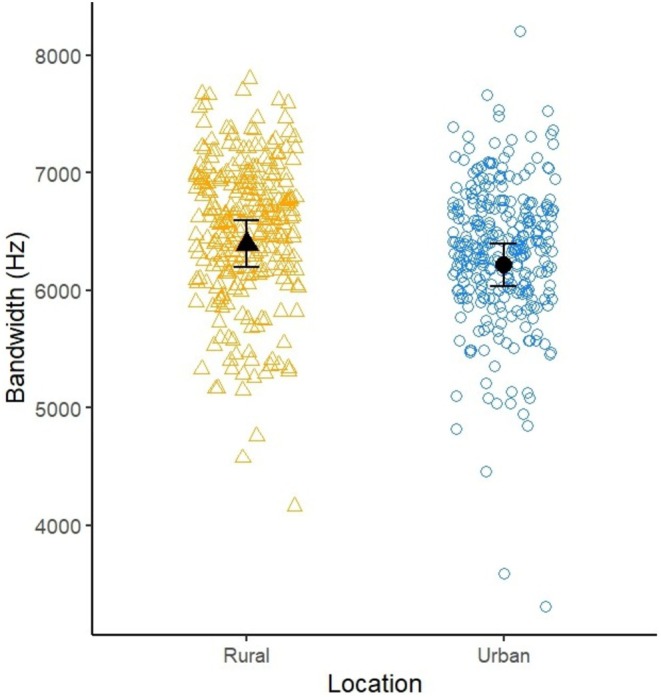
Scatterplot of the frequency range of rural (R; orange triangles) and urban (U; blue circles) robins. Open datapoints are raw data, black datapoints are the mean ± s.e. estimated from the model.

Urban robins had a narrower frequency range compared to rural robins (rural—urban: 182 Hz, 95% CI [34.4, 330]; see Table 4; Figure 9).

**TABLE 4:** Output from a LMM investigating the effects of urbanisation on frequency range.

Model terms	Estimate	Std. Error	df	*t*	*p*
Intercept (Rural)	6395.694	195.785	103.128	32.667	< 2e‐16 ***
Urban	−182.285	74.563	102.298	−2.445	0.0162 *
Time	9.904	16.735	103.427	0.592	0.5553

Random terms	Variance
Robin ID	92,534
Residuals	260,050

*Note:* Data are from 572 songs recorded from 113 robins. Robin identity was fitted as a random term. Asterisks were reported as shown in the *R* summary view with: *p < 0.05. ***p < 0.001.

**FIGURE 10:**
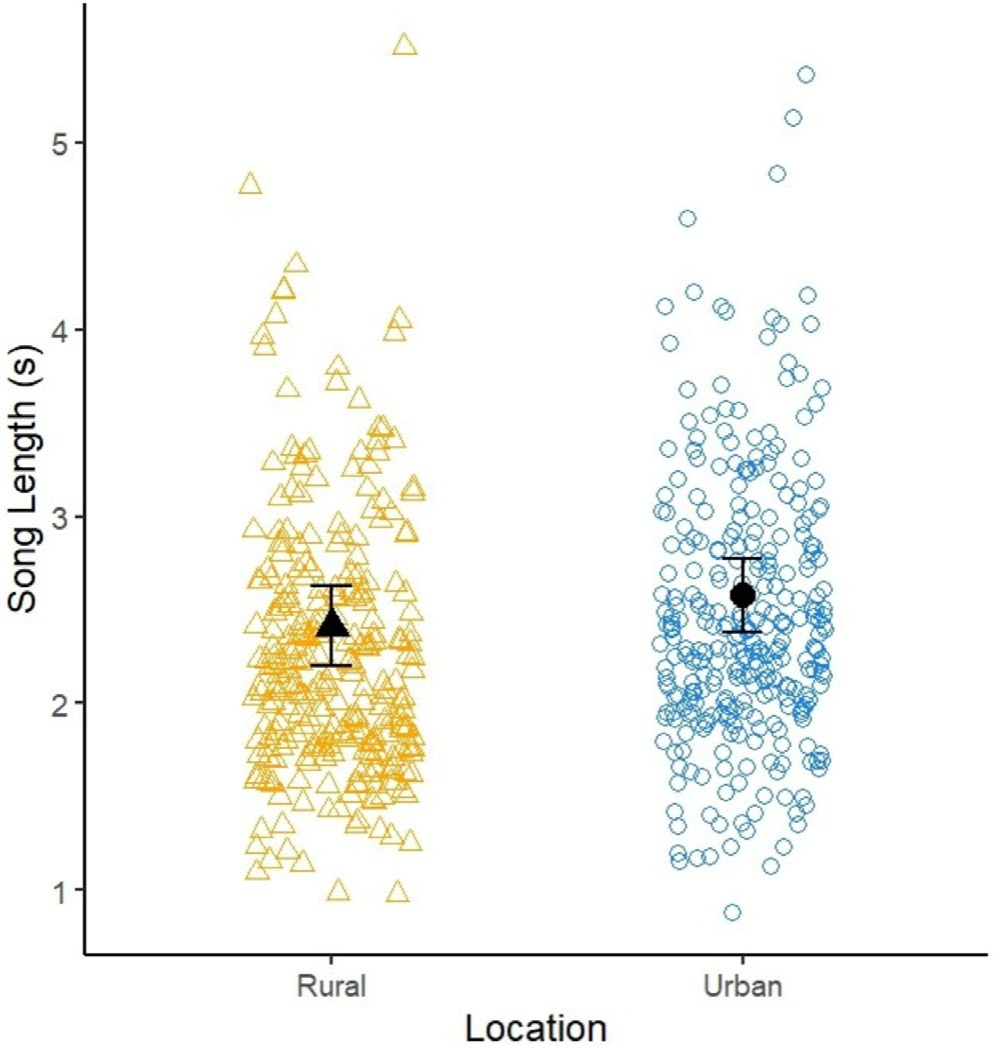
Scatterplot of the song length of rural (R; orange triangles) and urban (U; blue circles) robins. Open datapoints are raw data, black datapoints are the mean ± s.e. estimated from the model.

Urban robins had marginally longer songs (rural—urban: −0.159 s, 95% CI [−0.32, 0.00276]; see Table 5; Figure 10).

**TABLE 5:** Output from a LMM investigating the effects of urbanisation on song length.

Model terms	Estimate	Std. Error	df	*t*	*p*
Intercept (Rural)	2.41630	0.21386	104.16731	11.299	< 2e‐16 ***
Urban	0.15870	0.08140	103.33058	1.950	0.0539
Time	0.01146	−0.01828	104.40516	−0.627	0.5323

Random terms	Variance
Robin ID	0.08876
Residuals	0.41640

*Note:* Data are from 572 songs recorded from 113 robins. Robin identity was fitted as a random term. Asterisks were reported as shown in the *R* summary view with: ***p < 0.001.

**FIGURE 11:**
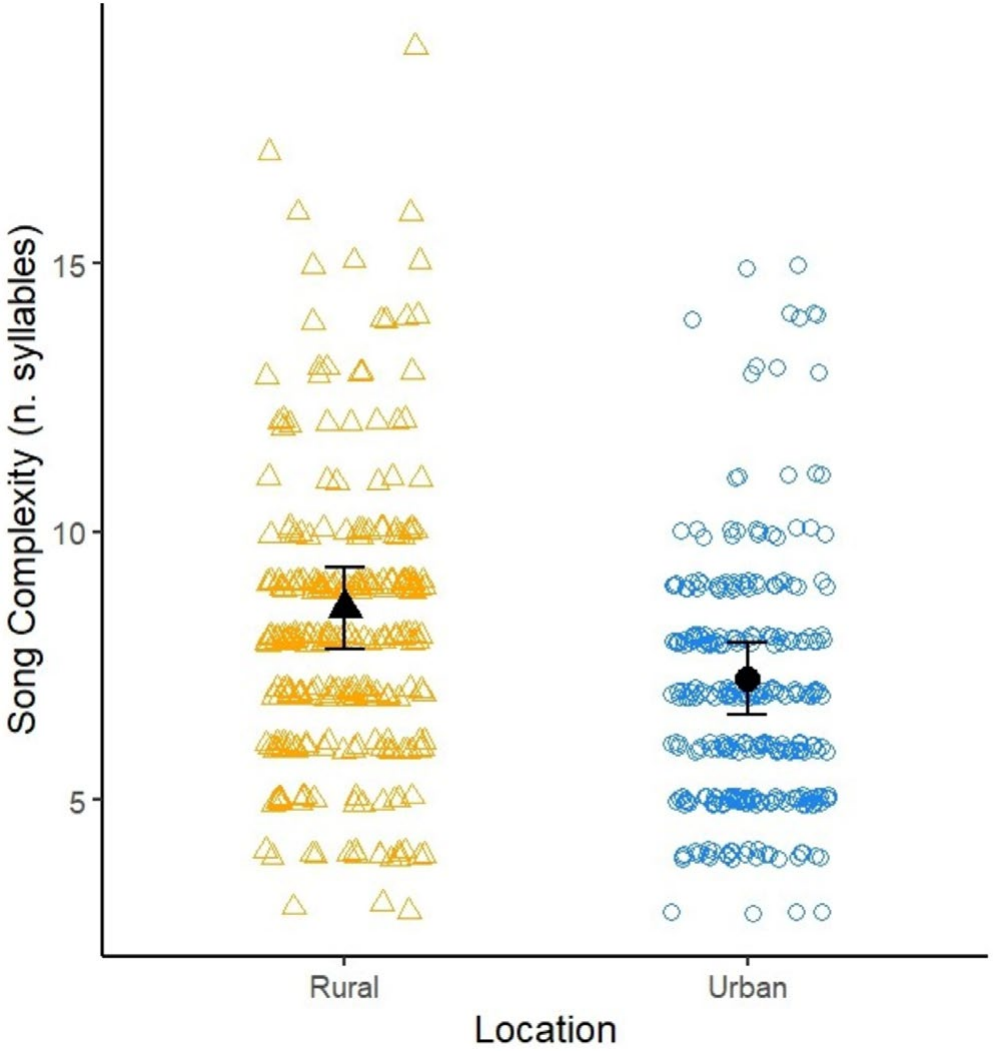
Scatterplot of the song complexity of rural (R; orange triangles) and urban (U; blue circles) robins. Open datapoints are raw data, black datapoints are the mean ± s.e. estimated from the model.

Urban robins had less complex songs, with a lower number of syllables (rural–urban: 1.32 syllables, 95% CI [0.753, 1.88]; see Table 6; Figure 11).

**TABLE 6:** Output from a LMM investigating the effects of urbanisation on song complexity.

Model terms	Estimate	Std. Error	df	*t*	*p*
Intercept (Rural)	8.57900	0.74913	110.13196	11.452	< 2e‐16 ***
Urban	−1.31895	0.28519	109.25620	−4.625	1.03e‐05 ***
Time	−0.03223	0.06404	110.41198	−0.503	0.616

Random terms	Variance
Robin ID	1.186
Residuals	4.628

*Note:* Data are from 572 songs recorded from 113 robins. Robin identity was fitted as a random term. Asterisks were reported as shown in the *R* summary view with: ***p < 0.001.

### 3.2 Re‐Recording Experiment

We re‐recorded and analysed 12 rural recordings, for a total of 130 up‐down songs. There was an upwards shift in the minimum frequency of 46.7 ± 74.4 Hz between rural re‐recordings without and with the urban background track, but this shift was not due to the difference between the background noise of the re‐recordings (no‐noise—noise: −46.7 Hz, 95% CI [−141, 48]; see Table 7).

**TABLE 7:** Output from a LMM investigating the difference in minimum frequency in rural recordings with and without urban background noise.

Model terms	Estimate	Std. Error	df	*t*	*p*
Intercept (No‐noise treatment)	2378.71	52.46	14.82	45.342	< 2e‐16 ***
Urban noise treatment	46.66	47.78	115.48	0.977	0.331

Random terms	Variance
Robin ID	18,443
Residuals	74,196

*Note:* Data are from 130 songs recorded from 12 robins. Robin identity was fitted as a random term. Asterisks were reported as shown in the *R* summary view with: ***p < 0.001.

## 4 Discussion

### 4.1 Responses to Anthrophony

The aim of this project was to look at how anthrophony affected the song of European robins. This was done by measuring and quantifying differences in song traits between individuals living in urban and rural environments.

#### 4.1.1 Shifts in Minimum Frequency

Urban robins had an overall higher minimum frequency compared to rural robins. The minimum frequency also showed a decreasing pattern in rural sites but remained constant in the city.

While many studies have documented the shift in minimum frequency in many bird species (Wood and Yezerinac 2006; Nemeth and Brumm 2009; Hu and Cardoso 2010; Potvin et al. 2011), including in robins (Montague et al. 2013), this is the first time, to our knowledge, that this diverging pattern has been observed. This might be because most studies have focused their sampling efforts on a small window of time in the morning rather than across the day (Nemeth and Brumm 2009; Hu and Cardoso 2010) or analysed minimum frequency shifts only in an urban setting (Wood and Yezerinac 2006).

In urban sites, persistently high traffic noise may constrain robins to maintain elevated minimum frequencies. On a shorter temporal scale of a few hours, robins have been observed to regulate in response to urban noise (Matheson 2025), as well as to experimental noise treatments (Montague et al. 2013). For this study, we did not attempt to measure ambient noise, nor did we differentiate between week and weekend days in the analysis, so it is possible that including these variables in future studies might show a finer regulation of minimum frequency across the day.

In rural sites, the higher minimum frequency in the morning hours could be seen as a response to biophony, particularly the dawn chorus, during which a high number of bird species sharing similar song ranges sing simultaneously (Gil and Llusia 2020). However, Malavasi and Farina (2013) showed that in dusk choruses, song frequency overlap and signal jamming are minimal, and that robins preferred to sing during refractory periods of other species, rather than simultaneously. This is consistent with the Acoustic Niche Hypothesis (Krause 1993), which suggests that birds regulate frequency and timing to minimise overlap between species.

In our early‐morning rural recordings, robin songs were partially isolated and aligned with refractory periods. However, this micro temporal shift might not have been sufficient to maximise signal transmission. Following the Acoustic Niche Hypothesis, it is theoretically possible that some species occupied the acoustic niche preferred by robins, pushing them to sing at a higher minimum frequency to properly maximise song transmission. As the day progressed, other species stopped or reduced their singing rate, allowing robins to return to their preferred acoustic niche.

Future studies could focus on characterising the acoustic niches distribution and occupation of this rural dawn chorus (as in Malavasi and Farina 2013), helping to better understand the acoustic structure of the avian community. Furthermore, Staniewicz et al. (2023) documented that robins sing both before and after the dawn chorus. It would be interesting to see if robins sing at a lower minimum frequency before dawn. If that were the case, it would provide additional support to the hypothesis that the rural increase of the minimum frequency was indeed in response to the dawn chorus.

Furthermore, in a natural environment robins often encounter noise sources other than biophony, such as rivers, the sea or the wind (geophony). Studies have shown that robins respond to geophony in a similar way as they do to anthrophony (Reed et al. 2022; Sebastianelli et al. 2022), by increasing minimum and dominant frequencies and reducing frequency range. Therefore, even though currently there is no specific evidence, it is reasonable to assume that robins regulate the minimum frequency in response to biophony as well.

#### 4.1.2 Changes in the Other Song Traits

The absence of a difference between the maximum frequencies in urban and rural robins is in line with what was found in other bird species (Wood and Yezerinac 2006). It can be explained by the presence of morphological constraints, such as body length, with which maximum frequency was found to be positively correlated (Liu et al. 2017).

The constraint in maximum frequency would also explain the wider range used by rural robins. With an environmental constraint at the lower end and a morphological constraint at the upper end, urban robins have a narrower range at their disposal. Thus, range may be influenced by the minimum frequency (Montague et al. 2013).

The longer song of urban robins is in contrast with what has been found in most previous studies (Nemeth and Brumm 2009; Montague et al. 2013, but see Hill et al. 2018). As seen in oriental magpie‐robins ( *Copsychus saularis* ) (Hill et al. 2018), European robins may also use longer songs to ensure a more efficient transmission of the most important parts of the song. Considering the marginal statistical outcome of the song length analysis, a more in‐depth analysis is suggested to better understand the song length response.

Despite being longer, urban songs contained fewer syllables (cf. Montague et al. 2013). They were mostly characterised by long notes, usually one or two per each up and down element, while rural songs had shorter and more elaborate notes for each up and down element.

Even though the complexity results align with the results of most studies (Nemeth and Brumm 2009; Montague et al. 2013), there are mixed findings about the effects of anthrophony on song complexity within the literature. Deoniziak and Osiejuk (2019) reported the opposite pattern in song thrushes, while other studies found that complexity does not change at all between urban and rural environments (Hill et al. 2018; Ríos‐Chelén et al. 2012; Brewer and Fudickar 2022). While keeping in mind that the species might partially explain the different results, it should also be noted that comparing song complexity analyses is challenging. There is no standardised method of assessment, and measurements have been made as either the number of notes, syllables or phrases within a song, or the number of syllable types within the whole song repertoire (MacDougall‐Shackleton et al. 2009; Brewer and Fudickar 2022).

#### 4.1.3 Plasticity of Song Traits

Despite the doubt that rural and urban song differ merely as a response to the structure of the two environments as predicted by the Acoustic Adaptation Hypothesis (Nemeth and Brumm 2009), rather than to anthrophony, there is growing evidence in support of the latter. Halfwerk and Slabbekoorn (2009) found an increase of minimum frequency with a play‐back experiment during which they exposed great tits to anthropogenic noise while still in their natural habitat. In more recent years, Stokstad (2020) recorded that in San Francisco urban birds were singing at lower frequencies once again as traffic noise drastically decreased during the COVID‐19 lockdown.

However, the two ideas are not mutually exclusive, and both factors (urban structure and noise) can simultaneously affect and shape bird song (Dowling et al. 2012; Job et al. 2016).

The evidence also supports that minimum frequency regulation is a plastic response, although the degree of flexibility is yet to be determined. The plasticity of this trait might be a partial reason as to why the robins have been able to adapt to an urban environment in the first place (Gross et al. 2010).

The variation in minimum frequency can have important effects on other song traits, such as the frequency range or the song complexity. Wood and Yezerinac (2006) showed that while minimum frequency varied with levels of background urban noise (also supported by Potvin et al. 2011; Matheson 2025), complexity did not. Nemeth and Brumm (2009) and Montague et al. (2013) both showed that there was indeed a distinct decrease in complexity in an urban setting, supporting the present findings. Montague et al. (2013) additionally showed that minimum frequency was negatively correlated to song complexity, supported by Winandy et al. (2021). It remains unclear whether the lack of more complex elements from urban songs is a direct response to anthrophony or a consequence of the increase in frequency and the subsequent range decrease and loss of low‐frequency elements (Montague et al. 2013), although the absence of a correlation between noise levels and song complexity (Wood and Yezerinac 2006) might suggest the latter. It has also been shown that a plastic response in one component constrains the expression of another trait. Particularly, Podos (1997) demonstrated that variations in frequency range affected trill rates in songbirds. Collectively, these findings indicate that minimum frequency plays a key role in shaping bird song and future studies should focus on better understanding its relationship with other song traits.

To cope with anthrophony, robins not only regulate minimum frequency, but also resort to temporal shifts and sing at night (Fuller et al. 2007). Given the variation in urban noise, monitoring songs over a full 24 h cycle could clarify the extent of temporal adjustment.

### 4.2 Fitness Consequences

Increasing the minimum frequency requires a greater muscle contraction at the syrinx (Suthers et al. 1999), which demands more energy. Despite having a possibly higher energy demand, urban robins sing longer. There is a distinct trade‐off between singing and foraging time (Gaunt et al. 1996), and the contradiction of spending less time foraging when the energy demand is potentially higher warrants a closer look in the future with direct measures of foraging behaviour or energy expenditure. Nonetheless, there might be additional variables, such as the increased signal transmission mentioned earlier, that are pushing urban robins to sing longer.

The changes seen in the analysed song traits could also have implications for the reproductive success of the robins, especially males, although this was not directly assessed in this study. Songs play a crucial role in the establishment and defence of high‐quality territories (Brindley 1991) and traits such as song complexity and repertoire often signal a male's health and experience (Hoelzel 1986; Catchpole and Slater 2008). Males with high‐quality, large territories have a higher chance of mating earlier compared to males with small territories (Harper 1985). It can be hypothesised that a decrease in song complexity, potentially constrained by increases in minimum frequency, may influence the fitness of urban robins, as low frequency elements are shown to be important in the defence against territory intruders (Zwart et al. 2016). Furthermore, proper territory defence might also be affected by the type of song robins choose, although it is still currently unknown if certain song elements or types, such as a trill or a slur, have a specific role within robin songs. If the constraints on urban robins prevent them from including key elements, the honesty of the song could potentially be affected, although this still remains to be tested. The reliability of the signal under anthrophony pressures could be maintained by female choice plasticity (Halfwerk, Bot, et al. 2011), as they learn to detect high‐quality males in the new noisy environment. This has already been found true in domestic canary females ( *Serinus canaria* ), which showed a decrease in low frequency song preference as urban noise increased (Des Aunay et al. 2014).

Although the observed differences between urban and rural songs are most likely driven by phenotypic plasticity, it can be hypothesised that two populations exposed to divergent acoustic environments over a long period of time, especially with no interaction between the two, could eventually lead to the genetic assimilation of song differences (Price et al. 2003; van der Burg et al. 2020; Vigne et al. 2021). This has already been found true in bananaquits ( *Coereba flaveola* ), with individuals from urban and rural environments presenting changes in genes associated with regulation of energetic metabolism and genetic expression (Mascarenhas et al. 2023).

### 4.3 Study Limitations and Future Research Directions

Despite the interesting results, any generalisations must be made with caution. Due to the nature of this project, we could not account for the sex or age of the recorded robins. Female and male territorial song differ, with female songs presenting more repeated elements and male songs being more complex (Hoelzel 1986). The two sexes could therefore be affected differently by anthrophony, and future studies could focus on such differences.

We also did not include all songs in the analysis, as the absence of mix and slur songs in some individuals reduced the accuracy of the models. A more detailed analysis could look for structural differences between song types (e.g., syllable structure and components), as well as how each type is affected by anthrophony. Furthermore, if a narrower frequency range facilitates trills (Podos 1997), future work should focus on why slur and mix song types, often characterised by trills, were sung less in the city. Additional constraints or variables could be at play when it comes to song choice. Future projects could also involve creating a proper repertoire database for urban and rural robin songs to better visualise inter‐ and intrasong variation. It would also allow not only for a more structured complexity analysis but also a deeper understanding of how specific syllables are used within songs. The constant presence of specific syllables in both environments may indicate the ones potentially important for signal reliability.

In summary, anthrophony has an important effect on multiple song traits of European robins. In particular, minimum frequency was affected not only by anthrophony for urban robins but also by time of day for rural robins, suggesting a response to biophony. Even though results showed that other traits also differed between urban and rural robins, it is unclear whether this is a direct response of anthrophony or a consequence of constraints of the shifts in minimum frequency on such traits.

Overall these results, particularly the increase of the minimum frequency, fit into the more general pattern arising across species globally (Slabbekoorn and Ripmeester 2008; Hu and Cardoso 2010; Seger‐Fullam et al. 2011; Ríos‐Chelén et al. 2012). The universality of the phenomenon shows the substantial impact that anthrophony is having on the avian community in an increasingly urbanised world.

## Author Contributions

**Marzia Golini:** conceptualization (lead), data curation (lead), formal analysis (equal), investigation (lead), methodology (lead), project administration (lead), visualization (lead), writing – original draft (lead), writing – review and editing (lead). **Matthew Bell:** conceptualization (supporting), data curation (supporting), formal analysis (equal), investigation (supporting), methodology (supporting), project administration (supporting), resources (lead), supervision (lead), validation (lead), visualization (supporting), writing – original draft (supporting), writing – review and editing (supporting).

## Funding

The authors have nothing to report.

## Conflicts of Interest

The authors declare no conflicts of interest.

## Data Availability

The data that support the findings of this study are openly available on Figshare: Recordings and annotation tables: https://doi.org/10.6084/m9.figshare.29916533. Dataset + readme file: https://doi.org/10.6084/m9.figshare.29916596. R code: https://doi.org/10.6084/m9.figshare.29916611.
